# Structural Insights into Iron Ions Accumulation in Dps Nanocage

**DOI:** 10.3390/ijms23105313

**Published:** 2022-05-10

**Authors:** Yury Chesnokov, Andrey Mozhaev, Roman Kamyshinsky, Alexander Gordienko, Liubov Dadinova

**Affiliations:** 1 Shubnikov Institute of Crystallography of Federal Scientific Research Centre “Crystallography and Photonics” of Russian Academy of Sciences, Leninskiy Prospect, 59, 119333 Moscow, Russia; chessyura@yandex.ru (Y.C.); a.a.mozhaev@gmail.com (A.M.); kamyshinsky.roman@gmail.com (R.K.); alex.gor99@mail.ru (A.G.); 2 National Research Center “Kurchatov Institute”, Akademika Kurchatova pl., 1, 123182 Moscow, Russia; 3 Shemyakin-Ovchinnikov Institute of Bioorganic Chemistry of Russian Academy of Sciences, Miklukho-Maklaya, 16/10, 117997 Moscow, Russia; 4 Faculty of Biology and Biotechnologies, National Research University Higher School of Economics, Myasnitskaya Str. 20, 101000 Moscow, Russia; 5 Institute of Translational Medicine, Pirogov Russian National Research Medical University, Ostrovitianov Str. 1, 117997 Moscow, Russia; 6 Moscow Institute of Physics and Technology, Institutsky Lane 9, 141700 Dolgoprudny, Russia; 7 Physics Department, Lomonosov Moscow State University, 119991 Moscow, Russia

**Keywords:** DNA-binding protein from starved cells (Dps), iron nanoparticles, Fe minerals, mini-ferritin, cryo-electron microscopy, single particle analysis

## Abstract

Dps (DNA-binding protein from starved cells) is well known for the structural protection of bacterial DNA by the formation of highly ordered intracellular assemblies under stress conditions. Moreover, this ferritin-like protein can perform fast oxidation of ferrous ions and subsequently accumulate clusters of ferric ions in its nanocages, thus providing the bacterium with physical and chemical protection. Here, cryo-electron microscopy was used to study the accumulation of iron ions in the nanocage of a Dps protein from *Escherichia coli*. We demonstrate that Fe^2+^ concentration in the solution and incubation time have an insignificant effect on the volume and the morphology of iron minerals formed in Dps nanocages. However, an increase in the Fe^2+^ level leads to an increase in the proportion of larger clusters and the clusters themselves are composed of discrete ~1–1.5 nm subunits.

## 1. Introduction

The DNA-binding protein from starved cells (Dps) interacts with DNA condensing, and compacting it into a higher ordered structure and protecting it from the destructive effects of high temperature, electromagnetic and UV radiation, as well as oxidative stress [1,2,3]. Dps is a multifunctional protein belonging to the ferritin superfamily, which includes prokaryotic and eukaryotic ferritins and heme-containing bacterioferritin and is called miniferritin [3,4,5,6]. While ferritins use molecular oxygen to catalyze Fe^2+^ oxidation, Dps utilizes hydrogen peroxide as a physiological iron oxidant, thus contributing to a decrease in the effectiveness of the Fenton reaction [7,8,9,10]. The formation of minerals in the ferritin cavity occurs in several stages: the penetration of Fe^2+^, the catalytic redox process with O_2_, the nucleation of Fe^3+^O and the growth of the mineral, and then the dissolution of minerals with the release of Fe^2+^. Moreover, the catalytic binding of 2Fe-/O_2_ occurs in ms, and nucleation/mineralization takes from minutes to hours. In eukaryotic ferritins, biomineral order/crystallinity is influenced by nucleation channels between active sites and the mineral growth cavity [10]. Ferritin can accommodate about 4500 iron atoms [11], forming nanominerals with diameter ~5 to ~8 nm [12]. Dps consists of 12 identical subunits and forms a dodecamer with 23 (tetrahedral) point group symmetry, which also has a hollow core and pores at the three-folds resulting in one of the smallest spherical protein cages that can accommodate up to 500 iron atoms [3,5,12,13,14]. In these nanocages Fe cations can be stored as oxyhydroxide clusters similar to ferritin [4]. The internal cavity, where iron is stored after oxidation, is connected to the external medium by hydrophobic and hydrophilic channels formed at the junction between the three-fold symmetry-related subunits. The hydrophilic channels are used by Fe^2+^ to enter the protein shell and reach the ferroxidase center located at the twofold symmetry interface [7]. Thus, Dps of *E. coli* has twelve ferroxidase centers each having two sites with different affinities for iron ions. The site with a higher affinity consists of His51 and His63 of one monomer. together with Glu82 of the adjacent monomer, and the site with low affinity is formed by Lys48 and Asp67 of one monomer in combination with Asp78 of the neighboring monomer [7]. Due to a crucial role of ferroxidase centers in survival of cells under oxidative stresses, the ability of Dps to bind DNA and accumulate iron was found in various types of bacteria [4,15,16,17,18,19,20,21,22,23,24,25,26,27,28,29,30,31,32,33]. Recently, we elucidated the effect of divalent iron cations on the structure of the protective Dps-DNA complex using small-angle X-ray scattering (SAXS) and cryo-electron microscopy (cryo-EM). It was found that the presence of Fe^2+^ in solution leads to the total destruction of the complex and aggregation. but the addition of EDTA as a chelating agent results in restoration of Dps-DNA complexes destroyed by Fe cations [34]. The dissolution of minerals Fe_2_O_3_·H_2_O occurs in response to the physiological requirements for iron in vivo [10].

Miniferritins Dps use H_2_O_2_ to produce ferritin mineral, thereby resisting the antibacterial H_2_O_2_ released by the host. Currently, miniferritins of human pathogens have been targeted as antigens in vaccines. However, even though Dps is associated with bacterial virulence, the idea of affecting bacteria by inhibiting the catalytic functions of their miniferritins is unpopular. Protein nanocages can also be used for constructing robust and configurable therapeutic delivery vectors [35], vaccines [36], nanoreactors [37] and templates for the synthesis of diverse nanomaterials [6,38,39,40,41].

Historically, ferritin core formation has been extensively studied using various Transmission Electron Microscopy (TEM) and analytical spectroscopy methods [42]. Single particle analysis of HAADF STEM (high angle annular dark field scanning transmission electron microscopy) images demonstrated that the mineral core of human hepatic ferritin has polycrystalline structure and consists of several ~2 nm subunits [43] and the obtained 3D reconstruction of an ‘average’ core reflects the cubic symmetry of the protein shell. In [44] graphene liquid cell-TEM (GLC-TEM) was used for in situ characterization of the iron oxide mineral cores in human heart ferritins (HHFs) and human spleen ferritins (HSFs) which exhibited different morphologies (possibly due to varying ratios of heavy and light subunits). 

However, for Dps, a deeper understanding of mineralization processes is needed. A detailed characterization of the different stages of iron cluster formation could contribute to a better understanding of the processes occurring in a nanocage, including whether the conformation of a mineral is regulated by external actors, etc., to stimulate the biotechnological use of miniferritins.

Given the recent technological advances in the field of structural biochemistry and biotechnology and as a continuation of our previous research [34], herein we present the results of cryo-EM single particle analysis (SPA) study of the iron ions accumulation process in the nanocage of Dps protein.

## 2. Results and Discussion

While it is well established that the cavity of Dps can store up to 500 iron atoms [3,5,12,13,14], for detailed study of fast iron accumulation processes five concentrations of FeSO_4_ (50, 200, 350, 500 and 2000 iron atoms per Dps dodecamer) were used. The prepared iron-containing samples are referred to in the following text as Dps-Fe50, Dps-Fe200, Dps-Fe350, Dps-Fe500 and Dps-Fe2000.

**Figure 1:**
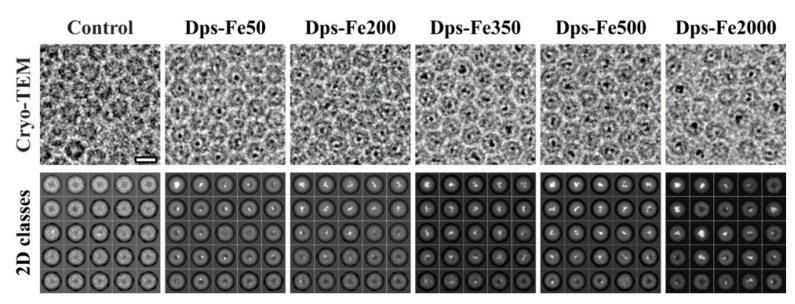
Typical cryo-EM images (1st line) and 2D class averages (2nd line) of Dps dodecamers with an estimated iron loading of 50–2000 atoms (left to the right) after 24 h incubation showing different morphologies of the iron clusters. Bar for cryo-EM images is 10 nm, each of 2D class averages is 12.5 nm wide.

Figure 1 demonstrates cryo-EM images and 2D class averages of Dps particles upon the addition of FeSO_4_ to the mixture and 24h incubation. Dark spots on cryo-EM images indicate the formation of the iron clusters inside the Dps cavities (Figure 1, 1st line). The obtained class averages suggest that the clusters (shown in white in Figure 1, 2nd line) are composed of discrete ~1–1.5 nm subunits.

**Figure 2:**
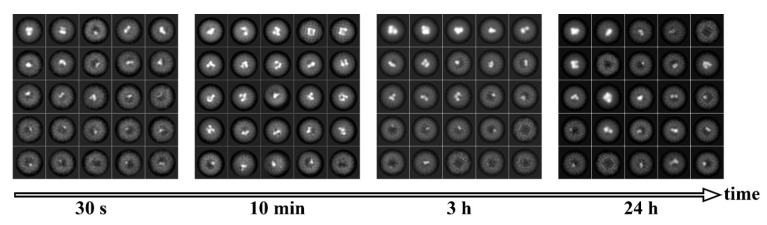
Class averages of Dps dodecamers with an estimated iron loading of 2000 atoms after incubation for 30 s, 10 min, 3 h and 24 h (left to the right). Each of 2D class averages is 12.5 nm wide.

**Figure A1:**
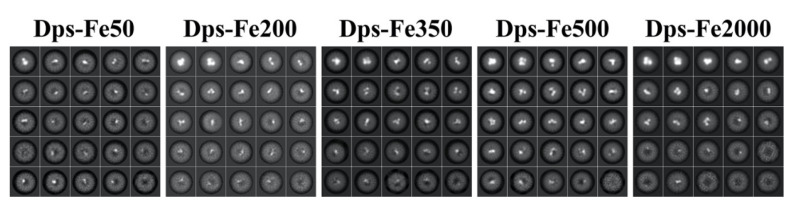
Class averages of Dps dodecamers with an estimated iron loading of between 50–2000 atoms (left to the right) after 3h incubation showing different morphologies of the iron cluster. Each of 2D class averages is 12.5 nm wide.

**Figure A2:**
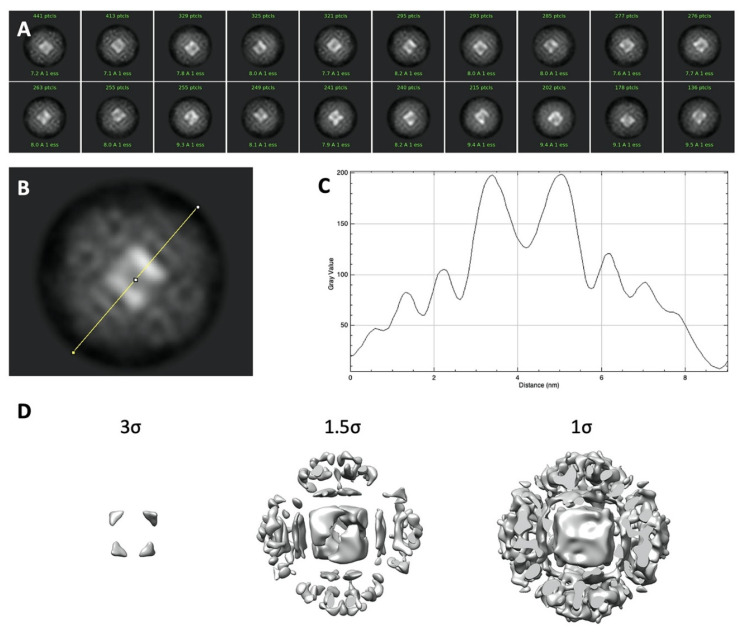
Formation of symmetrical mineral core in Dps-Fe2000 sample incubated for 10 min. (**A**) class averages (~9.5% of particles); (**B**,**C**) enlarged class average and corresponding intensity profile (measured along the yellow line), demonstrating low-density central region; (**D**) cryo-EM maps of Dps dodecamer with iron core at different thresholds (EMD-14952). Each of 2D class averages (**A**,**B**) is 12.5 nm wide.

All samples showed uneven filling of the Dps nanocages; even the mixture with the highest Fe concentration (Dps-Fe2000) demonstrated the presence of empty Dps particles. However, an increase in iron concentration leads to a distinguishable increase in the number of classes with a large (up to 30 nm^3^) core size. Such cores possess high contrast, which strongly affects the image alignment procedure, resulting in the lack of the features corresponding to the elements of the protein secondary structure on class averages. It is worth mentioning that the differences between 2D classes obtained from the samples incubated for 30 s, 10 min, 3 h or 24 h (see Figure 2 and Figure A1) are insignificant, which indicates that the cluster formation takes place almost immediately upon the addition of FeSO_4_ to the solution. Despite the high symmetry of the Dps dodecamer, the formation of the symmetrical mineral cores was observed for only 9.5% of the particles in the Dps-Fe2000 sample after 10 min incubation (Figure 2 and Figure A2). The morphology of such cores corresponded to a uniform hollow cube with thin walls (Figure A2, EMD-14952), differing significantly from all other samples but similar to the structure of hepatic ferritin [43].

**Figure 3:**
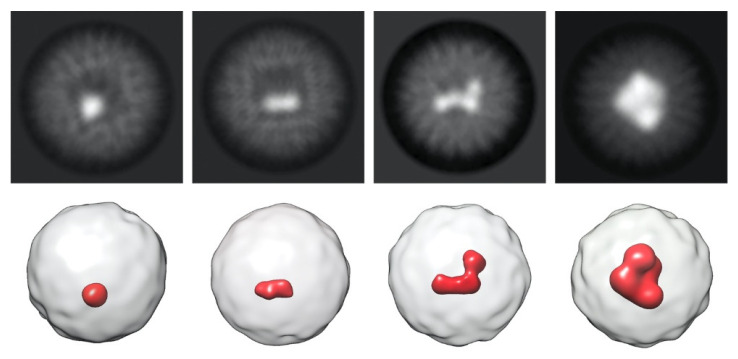
Cryo-EM class averages (**1st line**) and corresponding 3D reconstructions (**2nd line**) for different sizes of iron clusters (EMD-14948, EMD-14949, EMD-14950, EMD-14951). Dps is shown in white, Fe clusters are shown in red.

Further analysis was performed for the samples incubated for 24 h. To determine the morphology of iron minerals, ab initio models were generated using the stochastic gradient descent (SGD) method followed by 3D classification of single particles. The smallest mineral observed was approximately 1 nm in diameter and had a spherical shape, as described previously [34]. Figure 3 demonstrates diverse morphology of the iron minerals at different levels of Dps cavity filling (EMD-14948, EMD-14949, EMD-14950, EMD-14951). The accumulation of iron atoms occurs heterogeneously in the cavity of the Dps dodecamer, which leads to a displacement of the mass center from the center of the nanocage.

**Figure 4:**
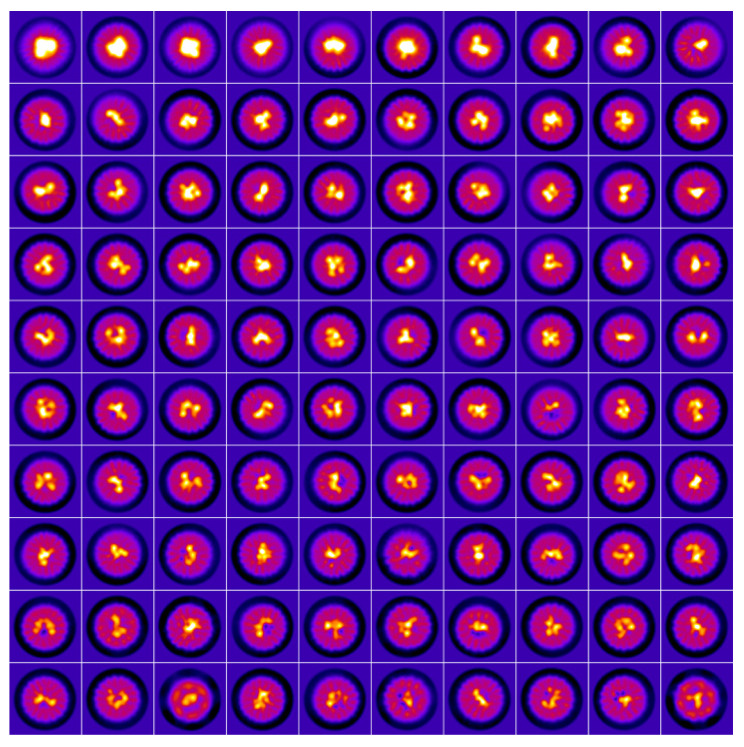
Cryo-EM class averages demonstrating various morphologies of the iron cluster inside the Dps. Merged dataset from all the samples under study. Dps dodecamers with empty cavities as well as with the smallest (~1 nm^3^) iron clusters are excluded.

Figure 4 shows a wide variety of mineral core projections, which can be described using the terminology introduced in [45] for classification of the ferritin iron core morphology based on 2D HAADF STEM (High Angle Annular Dark Field Scanning Transmission Electron Microscopy) images as ‘small circle’, ‘dumbbell’, ‘crescent’, ‘doughnut’ and ‘full raft’. The majority of the clusters consist of subunits, except for those with a ‘full raft’ morphology, and the morphology changes with iron loading, which was previously shown for ferritin using HAADF STEM [43] and GLC-TEM [44].

**Figure 5:**
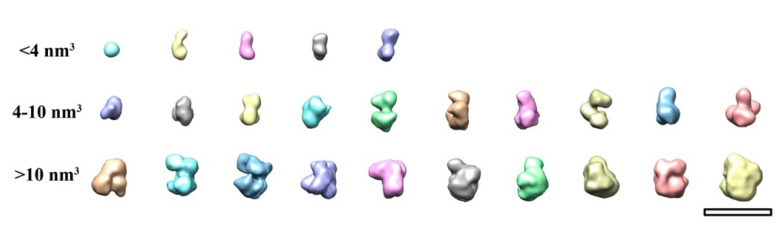
3D classes demonstrating morphological diversity of iron clusters in Dps. The classes are arranged in ascending volume (left to right). Scale bar is 5 nm.

However, in contrast to the HAADF STEM approach, cryo-EM in the defocus mode does not allow for unequivocal determination of the amount of Fe atoms in the cluster using intensity. To compare relative volumes of the iron minerals using cryo-EM SPA the intensity threshold equal to three protein intensity values was chosen, which ensured both the absence of densities from the Dps and the comparability of the iron cluster sizes to the 2D projections on class averages (Figure 5). 

**Figure A3:**
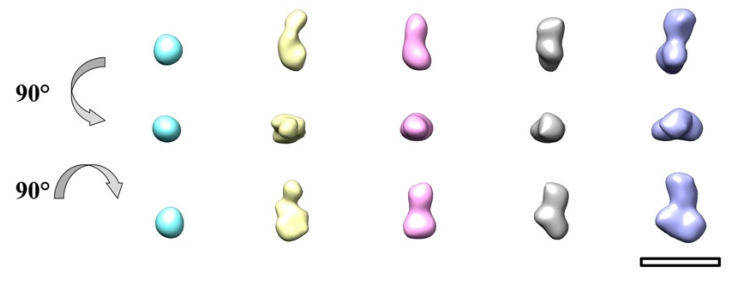
Various projections of iron clusters with a volume less than 4 nm^3^ at a threshold of 3σ divided into 5 3D classes. The classes are arranged in ascending volume (left to right). Scale bar is 5 nm.

**Figure A4:**
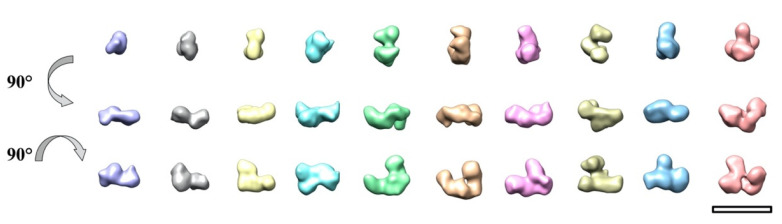
Various projections of iron clusters with a volume in the range 4–10 nm^3^ at a threshold of 3σ divided into 10 3D classes. The classes are arranged in ascending volume (left to right). Scale bar is 5 nm.

**Figure A5:**
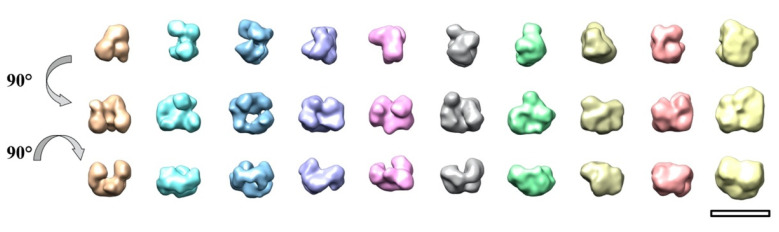
Various projections of iron clusters with a volume in the range 10–30 nm^3^ at a threshold of 3σ divided into 10 3D classes. The classes are arranged in ascending volume (left to right). Scale bar is 5 nm.

The cluster with the ‘small circle’ morphology has a volume <1.5 nm^3^, the size of the cluster with ‘dumbbell’ morphology is in the range from 1.5 nm^3^ to 4 nm^3^ (see Figure 5 and Figure A3). Minerals with 4–10 nm^3^ volume consist of several contacting subunits lining the inner surface of the Dps cavity, which leads to a large variety of the observed morphologies (see Figure 5 and Figure A4). Nuclei larger than 10 nm^3^ are characterized by greater homogeneity (absence of subunits due to fusion) and their various projections can correspond to the ‘crescent’, ‘doughnut’ and ‘full raft’ morphologies (Figure 5 and Figure A5).

**Figure 6:**
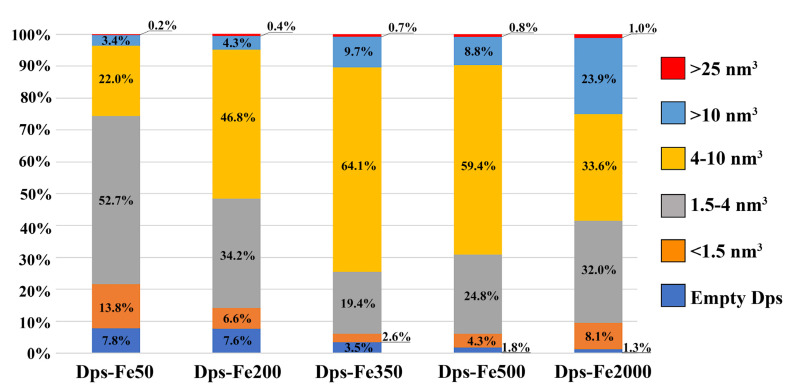
Histograms of the iron core volume distribution in various samples incubated for 24 h.

It was found that an increase in the iron concentration leads to an increase in the percentage of clusters with a larger volume; the results of the 3D classification are presented in the histogram in Figure 6. Despite the high symmetry of the Dps dodecamer, the formation of the symmetrical minerals was not observed (one could expect tetrahedral nuclei with vertices near the pores). In this case, the process of mineral formation can proceed by several reactions. The primary catalytic reaction mediated by miniferritins occurs at the ferroxidase centers. By using either hydrogen peroxide or molecular oxygen as electron acceptors, the Fe^2+^ ions that reach the ferroxidase centers at the dimer interface are oxidized to Fe^3+^ before moving to a nucleation site to start the mineralization process.

In our experiments, the co-substrate was oxygen and, accordingly, the ferroxidation reaction [46]:

[Fe2 − Dps]Z+2 + ½O2 + H2O → [Fe2(OH)2 – Dps)]Z + 2H+

where [Fe_2_ – Dps]^Z+2^ represents a dinuclear iron center and Fe_2_(OH)_2_–Dps represent the oxidized iron species.

After ferroxidation, ferric species leave the ferroxidase centers towards the carboxylate-rich lining of the inner cavity, where the ferrihydrite mineral core is formed. Following the initial iron turnovers by the apo-form of the protein, di-ferric oxo-species participate in a nucleation reaction that occurs in putative specific sites. The subsequent chain of ferroxidation and mineralization reactions leads to the formation of the ferrihydrite mineral core that grows from the initial nucleation sites [14].

The chemical composition of the mineral core does not depend on the co-substrate used in the iron oxidation reaction; in our study, the chemical equation for the reaction of mineralization with molecular oxygen [46,47,48]:

4Fe^2+^ + O_2_ + 8H_2_O → 4FeOOH_(s)_ + 8H^+^

where FeOOH represents the iron(III)oxide-hydroxide that composes the ferrihydrite mineral.

We can also assume that there could be a lack of molecular oxygen in the protein solution, in which case some part of the core was formed by the reaction:

Fe^2+^ + H_2_O + Cl^−^ → FeOHCl_(s)_ + H^+^

Thus, in the present study, cryo-EM SPA was successfully applied to explore the morphological diversity of the iron clusters formed in Dps nanocages. It was established that iron clusters have an inhomogeneous structure consisting of discrete subunits ~1–1.5 nm in diameter. The formation of the mineral occurred in a matter of seconds close to the inner surface of Dps cavity and further incubation up to 24 h did not lead to any significant changes. Our study proves that the entry of Fe^2+^ into the Dps cavity occurs spontaneously in solution, similar to that of ferritin [10]. Moreover, it was found that the increase in the incubation time and Fe^2+^ concentration does not affect the morphological diversity and volume distribution of the clusters. While the obtained results confirm the literature data that Dps loading does not exceed 500 Fe atoms per dodecamer [3,5,12,13,14], we have shown that even at concentration of 2000 atoms per dodecamer the cavities of most Dps particles are only partially filled with iron minerals. This is possibly due to the presence of EDTA in the buffer, which is capable of forming salts of ethylenediaminetetraacetates with metal cations. It was demonstrated that the filling of the nanocage occurs unevenly and an increase in the concentration of iron in the solution results in an increase of the average size of the iron cluster.

## 3. Materials and Methods

### 3.1. Preparation of Fe-Containing Dps Samples

Expression, purification and analysis of *E. coli* Dps was performed as detailed in [49,50]. Freshly prepared FeSO_4_ (Merck Millipore, Burlington, MA, USA) was added to a 3 mg/mL Dps solution in 50 mM NaCl, 0.5 mM EDTA, 50 mM Tris-HCl pH 8.0 in an amount corresponding to 50, 200, 350, 500 and 2000 iron atoms per dodecamer protein. Incubation was carried out at room temperature for 30 s, 10 min, 3 h or 24 h.

### 3.2. Cryo-Electron Microscopy

An amount of 3 µL of the Dps-Fe solution mixture was applied to a Lacey EM grid (Ted Pella, Northport, NY, USA) glow discharged for 30 s at 0.26 mbar pressure using current of 25 mA with Pelco EasiGlow (Ted Pella, Northport, NY, USA). The grids were then blotted with filter paper for 2.5 s from both sides at T = 20 °C and vitrified using Vitrobot Mark IV (Thermo Fisher Scientific, Hillsboro, OR, USA). Cryo-EM study was conducted using Titan Krios (Thermo Fisher Scientific, Hillsboro, OR, USA) equipped with Falcon 2 direct electron detector (Thermo Fisher Scientific, Hillsboro, OR, USA) and Image Corrector (CEOS, Heidelberg, Germany) operated at 300 kV. Images were obtained using EPU software (ThermoFisher Scientific, Hillsboro, OR, USA).

For SPA, movies of the Dps-Fe samples were obtained at 75,000× with 0.86 Å pixel size. Each movie consisted of 40 frames and was collected for 2 s. Total dose per movie was ~80 e/Å^2^; defocus values were in the range of 0.8–2.0 µm. Drift correction, CTF and defocus estimation, particle picking and extraction were conducted with Warp [51]; further processing was performed in CryoSPARC [52].

After the 1st round of 2D classification 267,406 particles of Dps-Fe50 sample (from 742 movies), 75,418 particles of Dps-Fe200 (from 108 movies), 321,356 particles of Dps-Fe350 (from 271 movies), 110,412 particles of Dps-Fe500 (from 164 movies), and 47,625 particles of Dps-Fe2000 (from 134 movies) were selected. Particles without Fe-containing core were removed after 2D classification and particles with iron core from all the samples were pooled into one set for further analysis. For this set 10 ab initio models were obtained and hetero refinement procedure was performed. The resolution of the obtained reconstructions was in the range of 7–10 Å (FSC = 0.143 criteria). Since alpha helices were not distinguishable on most reconstructions, it was impossible to determine the exact orientation of the core relative to the Dps dodecamer.

**Figure 7:**
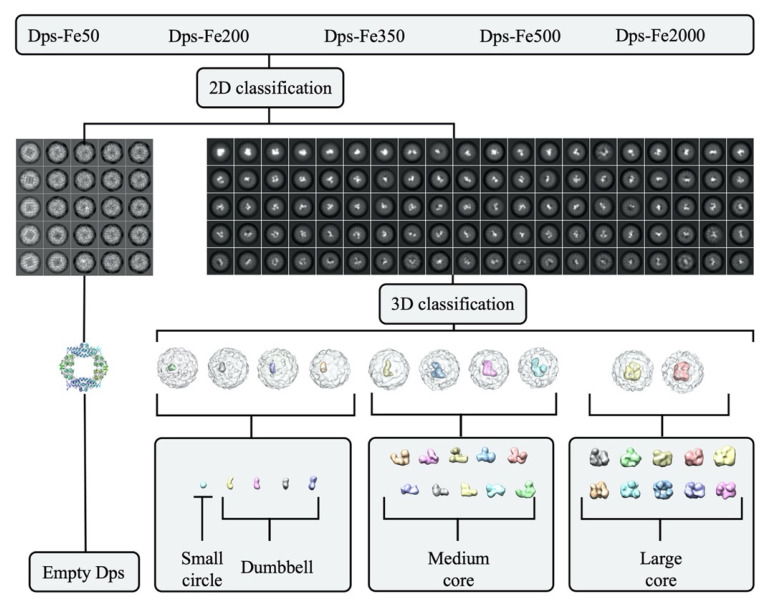
Cryo-EM data curation scheme.

The resulting models were sorted by the core volume, the shape of the core was examined visually. For these density maps a mask was applied to the protein and the average intensity from the protein was determined; to determine the volume of the nucleus, a threshold was chosen equal to three protein intensity values (due to high contrast of the nucleus), at which no protein was observed for all reconstructions, and the sizes of the nuclei were comparable to those in 2D classes. Overall data curation procedure is shown in Figure 7.

The obtained density maps were divided into three groups depending on the volume of the nucleus: V_1_ < 4 nm^3^, 4 nm^3^ ≤ V_2_ ≤ 10 nm^3^, V_3_ > 10 nm^3^. The group V_1_ was divided into five 3D classes; groups V_2_ and V_3_ were divided into 10 classes each. The classification results are shown in Figure 5, Figure A3, Figure A4 and Figure A5. The obtained metadata containing information about the number of particles from a particular sample in a particular class was used to build a histogram (Figure 6) of the iron core volume distribution.

**Figure A6:**
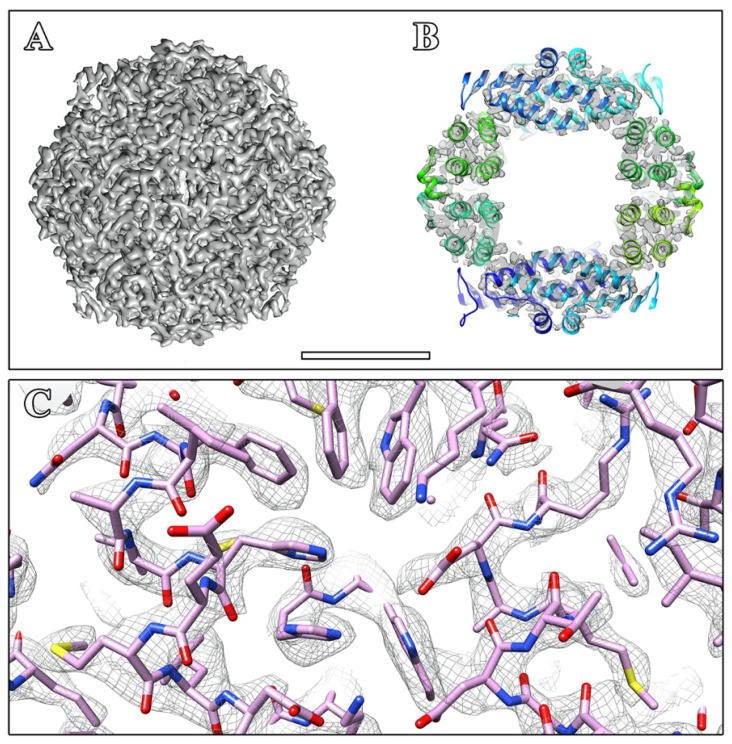
Cryo-EM map of Dps. (**A**) Dps surface; (**B**) slice through the center of Dps, PDB 1DPS; (**C**) high resolution cryo-EM map (EMD-14943) demonstrating ferroxidase center. Scale bar (**A**,**B**) is 4 nm.

For the control Dps sample, 515 movies and 414,851 particles were obtained. After 2D classification, 246,751 Dps particles with an estimated resolution above 4 Å were selected for further processing. These particles were used to obtain ab initio model (using P23 symmetry) and 3D reconstruction of Dps dodecamer with a resolution of 3.16 Å. The subsequent 3D classification allowed the selection of 131,127 particles for the final reconstruction by the non-uniform refinement method, resulting in a 2.59 Å density map (using P23 symmetry; see Figure A6). The obtained cryo-EM map was deposited to EMDB under the code EMD-14943.

## 4. Conclusions

The Dps proteins are expressed in bacterial cells under nutritional and oxidative stress conditions to protect DNA. Since Dps is a key component of the protective Dps-DNA, on which the efficiency of bacterial protection depends, we investigated the process of formation of the iron minerals inside the Dps nanocage. Herein, for the first time, it was determined that the formation of mineral core in Dps nanocage occurs within 30 s, with no significant changes being observed within the next 24 h. It was found that the size and morphology of iron clusters essentially do not depend on the concentration of iron ions in solution, and the majority of clusters consist of discrete 1–1.5 nm nanoparticles. The obtained results can be useful for nanoparticle synthesis and practical applications in biotechnology.

## Data Availability

All data are available in the manuscript, electron microscopy density maps are deposited in the EMDB (EMD-14943, EMD-14948, EMD-14949, EMD-14950, EMD-14951, EMD-14952).
